# Exploring Multiplex Immunohistochemistry (mIHC) Techniques and Histopathology Image Analysis: Current Practice and Potential for Clinical Incorporation

**DOI:** 10.1002/cam4.70523

**Published:** 2025-01-07

**Authors:** Aria Kaiyuan Sun, Song Fan, Siu Wai Choi

**Affiliations:** ^1^ Department of Anaesthesiology, School of Clinical Medicine, Faculty of Medicine The University of Hong Kong Hong Kong Hong Kong; ^2^ Guangdong Provincial Key Laboratory of Malignant Tumor Epigenetics and Gene Regulation Sun Yat‐Sen Memorial Hospital Guangzhou China; ^3^ Department of Orthopaedics and Traumatology, School of Clinical Medicine, Faculty of Medicine The University of Hong Kong Hong Kong Hong Kong

**Keywords:** cancer, histopathology, IHC, mIHC, multiplex immunohistochemistry, tumour biomarkers

## Abstract

**Background:**

By simultaneously staining multiple immunomarkers on a single tissue section, multiplexed immunohistochemistry (mIHC) enhances the amount of information that can be observed in a single tissue section and thus can be a powerful tool to visualise cellular interactions directly in the tumour microenvironment. Performing mIHC remains technically and practically challenging, and this technique has many limitations if not properly validated. However, with proper validation, heterogeneity between histopathological images can be avoided.

**Aims:**

This review aimed to summarize the currently used methods and to propose a standardised method for effective mIHC.

**Materials and Methods:**

An extensive literature review was conducted to identify different methods currently in use for mIHC.

**Results:**

Guidelines for antibody selection, panel design, antibody validation and analytical strategies are given. The advantages and disadvantages of each method are discussed.

**Conclusion:**

This review summarizes widely used pathology imaging software and discusses the potential for automation of pathology image analysis so that mIHC technology can be a truly powerful tool for research as well as clinical use.

## 1 Introduction

Immunohistochemistry (IHC) technology, used to detect and localise the expression of specific proteins in tissues, has been widely used to investigate the biological features present in the microenvironment. It provides valuable information for cancer research, playing an important role in guiding subsequent clinical treatments [1].

However, traditional single‐color IHC lacks the ability to capture the biological complexity between cells and is not able to reflect the geographic features of the tumour microenvironment. The development of brightfield multiplexed immunohistochemistry (brightfield mIHC) has solved certain issues relating to visualisation, but is still limited by relatively low sensitivity for protein detection and limited by the broad spectral absorption of chromogens. This broad spectral absorption may cause signal overlap or interference, posing difficulties in achieving the desired levels of specificity and sensitivity in target detection. In recent years, novel mIHC technologies have overcome these limitations by utilising fluorescent tags and advanced imaging techniques, providing a more convenient way to obtain precise information on the expression and simultaneous localisation of multiple proteins in tissues [2, 3, 4].

Novel fluorescence‐based mIHC allows a wider range of applications. In addition to the ability to simultaneously detect multiple biomarkers, mIHC enables the detection of the signals of some low‐abundance proteins, which allows mIHC to detect more complex cell subpopulations, characterise immune responses, assess tumour heterogeneity and evaluate therapeutic targets [5, 6, 7, 8]. Therefore, mIHC offers valuable insights into the spatial relationships between cells within complex tumour microenvironments (TMEs) [9, 10, 11, 12]. Furthermore, mIHC has potential in the clinical arena to provide a rationale for grouping patients for treatment and data for identifying cancer patients with a higher risk of metastasis [13].

Immunohistochemistry (IHC) is a powerful technology in the field of biomedical research and clinical diagnostics. In this review, applications and advances made in mIHC will be discussed, and technical details including antibody selection, imaging modalities and data analysis methods will be summarised. Different applications of mIHC in various research domains and its potential impact on clinical practice will be assessed.

## 2 Comparison of mIHC Versus Singleplex Staining

Single IHC is a conventional immunohistochemical technique for detection of a single protein target within a tissue slide, and is an essential tool for pathological diagnosis and research. On the other hand, mIHC allows for the simultaneous detection of multiple protein targets in a single tissue slide, providing a comprehensive perspective of protein expression and interaction. The choice between these two techniques depends on the research requirements, the number of protein targets of interest and the complexity of the microcosm under study.

Tumour progression is a multifactorial process and as such, must be influenced by the interplay of many proteins both in the micro‐ and the macro‐environment. In this context, the traditional single IHC is not sufficient when more complex information is needed to provide support for mechanistic hypotheses [14]. Therefore, mIHC, which can detect multiple protein targets simultaneously on the same tissue slide, can streamline the detection process while providing information on the spatial aspects of protein expression [15].

A single biomarker can only provide a single piece of information. In contrast, the knowledge gained from assessing multiple biomarkers interacting with each other can be more helpful in understanding the complex interplay between these proteins. Also, biomarkers are sometimes of better predictive value when analysed in combination, such as this example demonstrated in a study on pancreatic cancer, where the localisation of multiple signals was detected in tissue microarrays (TMA) slides. This study was able to demonstrate that the group of B7‐H3, B7‐H4 and HHLA2 is associated with the prognosis of pancreatic cancer patients [16]. Another study similarly validated that tumour‐infiltrating CD8^+^ T cells can be identified through the biomarker groups CD8, CD3, forkhead box P3 (FOXP3) and CD20 expression [17]. These studies show that probing the complexities of coexpression and spatial distribution in the tumour microenvironment can improve knowledge of disease mechanisms and progression.

Additionally, using mIHC to visualise multiprotein targets detected in single tissue sections can also help to visualise the spatial distribution of cells in the tumour microenvironment in an intuitive manner, and information can then be analysed for more meaningful and clinically relevant information [15, 18, 19].

Besides providing detailed information which is not possible with single IHC, mIHC also confers the advantage of being more cost‐effective and time‐efficient than multiple rounds of single IHC, enabling a single experiment to analyse multiple protein targets and reducing the consumption of reagents, slides, as well as laboratory resources.

To improve efficiency, and to complement guided diagnosis and therapy, the simultaneous evaluation of multiple targets will become of utmost importance in tumour research [20].

## 3 Technical Overview of Multiplex IHC Staining

### 3.1 Conventional mIHC (Brightfield Multiplexing) Versus mIHC (Tyramide Signal Amplification)

Conventional methods for developing multiple colours in mIHC are complicated and restrictive. To avoid cross‐reactivity when labelling secondary antibodies in multiplexed IHC on the same tissue section, the conventional chromogenic mIHC method usually involves alternating labelling with mixtures of primary antibodies derived from different species or isoforms (such as mouse, rabbit or goat) [21, 22]. Then, secondary antibodies specific to the primary species are used, followed by staining with different dyes, such as DAB (3,3′‐diaminobiphenyl) and Fast Red. In the antigen–antibody reaction, the antigen to be detected binds to a specific antibody, this specific antibody will then bind to an enzyme‐labelled secondary antibody then binds. Dye is added to the sample and pigments react with the enzyme so that the antigen and antibody can be visualised and information of their position can be obtained [21, 22].

However, this staining approach has significant drawbacks. Firstly, the selection of antibodies from multiple species is complex and requires a more sophisticated experimental design to choose the appropriate antibody for the experiment. Furthermore, most commercially available antibodies are produced in rabbits due to simplicity and cost‐effectiveness. It also becomes challenging to distinguish different chromophores on slides, especially when there is overlap in colour between two chromophores. There are relatively few chromophores available for brightfield microscopy compared to fluorescent dyes, and their absorption spectra are broader than those of fluorophores, which means that spectral overlap between chromophores is likely to occur in the brightfield [23, 24, 25].

**FIGURE 1:**
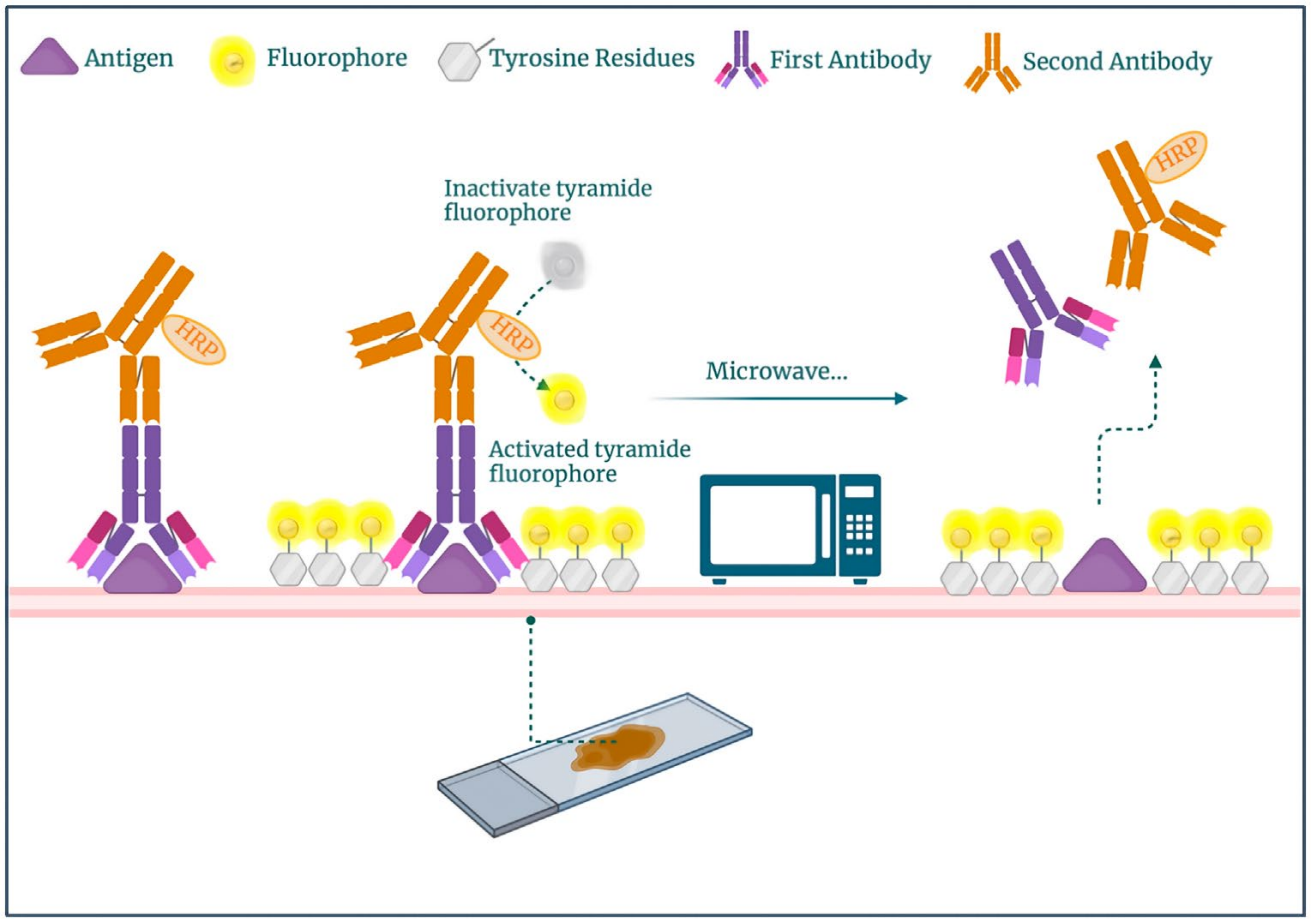
Mechanisms of tyramide signal amplification (TSA) in multiplex immunofluorescence staining.

Multiplex immunofluorescence staining utilising tyramide signal amplification (TSA) allows for the use of primary antibodies from the same species in a single staining set. In mIHC with TSA, a stripping step is incorporated, taking advantage of the permanent nature of the covalent tyramide–tyrosine bond (Figure 1). This enables the heat‐mediated removal of primary/secondary antibody signals without affecting the fluorescent signals. Consequently, antibody complexes can be stripped off, while the deposited fluorophore remains on the tissue surface [21, 24, 26, 27]. This approach eliminates the need to consider the host species of the primary antibody when selecting the target [21, 24]. Only the appropriate secondary antibody needs to be chosen based on the primary antibody specificity. This advancement has facilitated the development of mIHC experiments where antibody selection is based solely on performance criteria. The TSA technique can also be used for brightfield multiplexing, but capabilities are limited compared to fluorescence‐based multiplexing. TSA relies on chromogenic substrates to label the target antigen, resulting in different colours that can be easily distinguished from each other. But due to the limited number of chromogenic substrates in brightfield multiplexing, and some of the chromogenic substrates remaining on the tissue after heating, the use of TSA may not be useful in brightfield multiplexing.

However, the use of TSA in fluorescent dyes confers significant advantages in terms of its sensitivity and specificity in detecting signals compared to the brightfield dyes that are mostly used in traditional mIHC. These amplification techniques in mIHC can help researchers to detect proteins of lower abundance and improve the signal‐to‐noise ratio compared to traditional brightfield mIHC, thus improving the accuracy of protein quantification and analysis [24, 28].

#### 3.1.1 Signal Amplification

The tyramide signal amplification (TSA) technique utilises the properties of tyramide and enzymatic catalysis to enhance the detection signal of target molecules in immunohistochemistry [29, 30, 31].

In the TSA technique, horseradish peroxidase (HRP) and hydrogen peroxide are first added to the tissue sample. The hydrogen peroxide and the phenolic portion of tyramine react with horseradish peroxide to activate the free radicals formed on the tyramide substrate during the reaction [32]. The activated tyramine then covalently binds to nearby tyrosine residues in a covalent manner and further binds to nearby protein residues (tryptophan, histidine and tyrosine residues) to form stable fluorescent pairs. For optimal fluorescence amplification, tyramine is prelabelled with fluorophores [21].

The tyramide signal amplification technique increases mIHC sensitivity, providing clearer and brighter signals and making it ideal for identifying low‐expression target molecules.

### 3.2 Other Types of mIHC Techniques

#### 3.2.1 CODEX (Codetection by Indexing)

CODEX technology for codetection by indexing is a multistaining technique based on the principle of antibody labelling of fluorescent oligonucleotides using DNA‐coupled antibodies conjugated to dye‐labelled nucleotides [33]. Cells are stained with all labelled antibodies in the first instance and the proteins are then iteratively displayed and imaged as they are exposed to the nucleotide mixture [34]. The technology has recently been commercialised and the CODEX/Phenocycler allows the staining of fresh frozen tissue, single cells and FFPE tissue and can perform staining protocols with up to 60 markers on a single platform [34, 35]. In addition, the PhenoCycler is compatible with many existing inverted fluorescence microscopes, facilitating subsequent processing. However, use of the CODEX/PhenoCycler is limited due to high cost [36].

#### 3.2.2 Multiple Ion Beam Imaging (MIBI)

Multiple ion beam imaging (MIBI) technology supports the simultaneous detection of more than 40 proteins on a single tissue section with excellent spatial resolution [37]. Using the principles of multi‐isotope imaging mass spectrometry (MIMS), antibodies are labelled with metal chelating isotopes during incubation [38]. Heavy metal ions are then detected by secondary ion mass spectrometry (SIMS) to produce spatially resolved images. No fluorophores or enzyme‐linked reagents are used, and all targets are stained together in a single primary antibody premix, eliminating the need for sequential rounds of staining to introduce antibodies in batches, as is common in multiplex immunohistochemistry (mIHC).

After staining, samples are subjected to ion beam scanning, and the antibody labelled with the metal element is excited by secondary ions. The instrument extracts information about the target biomarker based on the antibody's unique metal isotope label [39]. This information is transferred to a time‐of‐flight mass spectrometer for identification and quantification, producing a multicolour image. By eliminating the lengthy fluorescence staining step, MIBI imaging technique avoids the interference of background noise caused by autofluorescence and produces a much clearer image signal than conventional mIHC [40].

#### 3.2.3 Tissue‐Based Cyclic Immunofluorescence (t‐CyCIF)

t‐CyCIF, which enables high‐dimensional multiparametric imaging, is also a powerful method for performing multiplexed tissue immunofluorescence. Introduced in 2018, this technique enables highly multiplexed immunofluorescence imaging, facilitating the identification of more than 60 different proteins in both normal and tumour human tissue samples [41]. It can analyse FFPE at the single cell level and is compatible with H&E staining, enabling the integration of fluorescence imaging into routine histopathological examinations. However, the process of multiple immunofluorescence staining can be time consuming and labour intensive. Furthermore, the challenges of validating and setting up a large number of antibodies are imperative considerations when implementing t‐CyCIF [41].

#### 3.2.4 Nanocrystal Quantum Dots

Quantum dots (QDs) are semiconducting nanocrystals with diameters typically in the range of 1–10 nm. Due to their small size and quantum effects, QDs exhibit unique optical and electrical properties that emit high‐intensity fluorescent signals, allowing QDs to be used as fluorescent probes for labelling a wide range of proteins of interest in multiplexed immunohistochemical (mIHC) assays [42]. Compared to conventional fluorescent dyes, QDs have a narrower emission spectrum and can be excited simultaneously by a single light source, resulting in less overlap between spectra. In addition, QDs have stable photochemical properties and the rate of fluorescence signal decay is slower than that of conventional fluorescent dyes. In mIHC applications, QDs are commonly used to label secondary antibodies for target detection [42].

#### 3.2.5 Multiplexed Immunohistochemical Consecutive Staining on Single Slide

Chromogenic IHC is also available in circular staining. It supports multiplexed immunohistochemical consecutive staining on single slide (MICSSS). Similar to traditional chromogenic IHC, MICSSS involves cyclic labelling, scanning and subsequent removal of the chromogenic substrate. Typically, each marker produces an individual immunostaining image of the entire tissue, and through iterative cycling, up to 10 markers can be displayed on an entire section [43].

However, a challenge is the potential loss of tissue antigenicity and integrity due to decolourisation and heat exposure during the cycling process. The extended duration of cyclic staining and the subsequent merging of images for analysis are also potential difficulties [43].

### 3.3 Different Antibody Stripping Methods

To prepare for a new round of staining, the previous round of antibodies must be removed. Currently, high‐temperature treatment is the primary method used for antibody stripping [21], and it is usually necessary to maintain this high temperature over a period of time [44]. The temperature required is time‐dependent, and to prevent large amounts of liquid from evaporating and causing the tissue sections to dry out, the duration of the heat treatment process needs to be controlled in practice according to the size of the vessel and the number of slices to be processed (normally > 100°C; 2–5 min) [45]. This method involves using a thermally mediated antigen retrieval solution that washes away the noncovalently bound antibodies from the previous round by boiling at a high temperature, while the covalently bound antibodies remain on the tissue. After the high‐temperature treatment, a new round of incubation is performed, with high temperatures used between each round of staining to strip away primary and secondary antibodies that have been added during each round of staining [21, 46].

However, there are certain considerations when using high‐temperature treatment. It is important to ensure that the tissue is heated evenly in the microwave oven during the antibody stripping process to obtain satisfactory experimental results. When using a microwave oven for antibody stripping, it is crucial to choose an oven that maintains a constant high temperature to ensure complete antibody stripping and to avoid potential signal interference [47].

Direct exposure of the sample to hot air during heated stripping operations can be detrimental. Constant high temperatures may cause the antigen retrieval solution to boil and evaporate faster, resulting in inadequate cleavage and direct exposure of the sample to high‐temperature air. This can lead to rapid drying of the sample, denaturation of proteins and cellular structures and ultimately damage to the sample. Therefore, it is critical to prevent dehydration of the sample by ensuring sufficient fluid volume to withstand evaporation at high temperatures and by monitoring the water level during the heating process [48].

Specific instruments made for staining, such as the Leica Bond or Ventana, are also used to perform the heating step in the staining process. These instruments are typically employed for automated immunohistochemistry and in situ hybridisation experiments, and their heating procedures are similar and usually include preparing the heating medium, setting up the staining programme, placing the samples, running the stainer and then observing the staining. These instruments allow researchers to perform the heating procedure with precision, accuracy and efficiency, greatly improving the quality of staining.

Another important aspect to consider is the choice of antigen retrieval solution [49, 50]. A commonly used protocol involves using a citrate buffer with a pH of 6.0 as the antigen repair solution. However, in the presence of a high pH antigen retrieval solution, the negative charge of the antigen molecules is increased, leading to increased adsorption of the antigen on the slide, and the antigen molecules are more likely to bind to the antigen on the slide, allowing the antigen to be more readily recognised and bound by the antibody, thereby producing a higher signal‐to‐noise ratio. Therefore, when multiple proteins are to be included in the staining protocol, it is important to adjust the reagents used based on the initial staining results for the different proteins to select the appropriate pH for antigen retrieval. Low pH antigen retrieval solutions are more suitable for multiple staining and can be used more than once between cycles, whereas high pH antigen retrieval solutions produce a higher signal‐to‐noise ratio.

### 3.4 Automated mIHC

Clinical researchers have increasingly recognised the significance of tissue analysis, as it reveals valuable, indicative information. While multiplex immunohistochemistry (mIHC) has improved efficiency and standardisation compared to traditional staining methods, the manual handling of numerous slides and the subsequent examination and analysis of stained sections remain labour‐intensive. Despite advancements, the manual procedures involved in staining multiple slides and analysing the stained sections are still time‐consuming and monotonous, requiring several days of intense labour for each staining round. Additionally, manual handling can never achieve complete standardisation, leading to variations in staining consistency. To utilise mIHC effectively as a diagnostic and prognostic tool, the automation and standardisation of staining and analysis procedures are necessary. In this regard, scientists have demonstrated that automated instruments can process up to 48 samples simultaneously, accomplishing the task in 15% less time compared to conducting the procedures manually. Furthermore, taking into account savings in labour and consumable costs, there is a 37.27% reduction in experimental costs when slides were labelled using automated immunostaining instruments [40]. Besides, the automated instrument allowed for 70 min of unattended time, saving both time and labour [51, 52].

Generally, the operation of an automated staining instrument only requires that the reagents be used according to the manufacturer's instructions. The operator only needs to prepare the required reagents according to the instructions and titrate them into the user‐fillable dispenser in order for the machine to run automatically. However, heating is still a key component of the automated procedure and so the temperature and duration of heating need to be adjusted accordingly in automated stainers.

## 4 Protocol for Multiplex Immunofluorescence Staining

Paraffin slides are placed on a hot plate to melt the paraffin, then hydrated in xylene baths and anhydrous ethanol baths. After hydration, heat treatment must be conducted: Slides are placed in a microwave oven and treated with an antigen retrieval solution (AR) at pH 9.0 (ethylenediaminetetraacetic acid buffer) or pH 6.0 (citrate buffer). High temperatures are applied at this point, and then kept at a medium–low heat to allow the solution to remain boiling for 15 min. The solution level must be monitored at all times to avoid drying out, and slides should not be directly exposed to high air temperatures.

After the heat treatment, tissues must undergo a sealing process using peroxidase. The first antibody is then applied and left to incubate for 1 h at a humidified environment or overnight at 4°C. After the first antibody incubation, the second antibody incubation is conducted and then fluorescent dye is applied. All steps are repeated after hydration for each subsequent round of staining with other antibodies and other chosen fluorescent dyes. When all antibodies to be stained have been applied, the antibodies are stripped again by heat treatment. The last step involves dripping DAPI onto the tissue. The type of specimen affects the rate of penetration and diffusion of the DAPI stain, and technically the optimum staining time can be determined by observing by eye the intensity and clarity of the staining of the nuclei.

## 5 Assignment and Compatibility of Antibodies in mIHC

Successful detection of proteins, that is, the primary antibodies, is crucial in protein detection and mIHC experiments. Monoclonal antibodies are usually preferred, and studies have shown that monoclonal antibodies have a higher specificity due to their unique properties against the target antigen's unique peptide, which is typically located in a region less affected by formalin fixation [53].

In addition to antibody selection, the appropriate concentration of the antibody is also a critical factor in the experiment's outcome. While manufacturers often provide recommended dilution concentrations for their antibodies, these concentrations do not guarantee the desired staining results. Antibody performance can vary greatly depending on the section type and experimental conditions. When performing multiple staining, the impact of multiple rounds of staining on previously stained antibodies must also be considered.

Therefore, it is advisable to conduct an antibody chromogenic assay at the initial stage of antibody use. Referring to past literature and the manufacturer's advice on antibody concentration, a basic IHC staining test should be performed. The results of this test serve as an important reference for determining the concentration and sequence of subsequent mIHC staining [54].

The sequence of mIHC staining should also take into account the condition of the antibodies and align with research requirements [9]. Firstly, the results of the chromogenic test should be considered [55, 56]. Antibodies with lower staining colour intensity should be adjusted and placed later in the sequence of multiple staining rounds, or their concentration should be appropriately increased. Conversely, antibodies with higher staining colour intensity should be placed earlier in the sequence. Secondly, the need for quantification should be considered. Antibodies requiring accurate quantitative results are best placed at the end of the sequence to minimise interference from repeated heat stripping and washing. Lastly, if the staining aims to explore spatial information in the tumour microenvironment rather than quantitative analysis, indicator cells (e.g., T cells, CD4, CD8) should be stained in the first step, while functional antigens serving as markers can be stained towards the end of the sequence to maximise retention of target information [56].

Although TSA‐based mIHC technology is well documented, it is recommended that researchers conduct antibody testing and signal elimination after thermal stripping of the antibodies used, based on their experimental needs, before proceeding with formal experiments. If some antibodies are not significantly removed after thermal stripping, potential interference with subsequent staining will occur. To avoid such interference, researchers should arrange their experiments based on the denaturation results of antibody detection, using antibodies that are less prone to stripping or incomplete denaturation for the final round of multiround staining. Proper ordering of antibodies enhances the accuracy of experimental results.

## 6 Imaging Approaches (Analytic Platforms for mIHC)

Scanning and imaging the sections with the help of specialised equipment is an essential step to visualise tissue sections after multiple protein labelling.

The Vectra Polaris is a widely used pathology imaging system that combines multispectral imaging with automated slide scanning, supporting up to 80 slides in a single operation. Other commonly used image acquisition and scanning instruments include the TissueFAXS SPECTRA and the Aperio Versa and the choice of which system is most suitable for the laboratory is dependent on the number of slides requiring processing and the space available to house the system.

After digitising, software with histological analysis capabilities is required to identify and classify cellular tissues accurately. Table 1 details some of the well‐established image analysis software.

**TABLE 1:** Image analysis software.

Name	Developed by	WSI processing capabilities	Open resource
InForm	PerkinElmer	Yes	No
HALO	Indica Labs	Yes	No
Qupath [55]	Bankhead et al.	Yes	Yes
Cellprofiler [56]	Broad Institute of MIT and Harvard	No	Yes
ImageJ [57]	National Institutes of Health	Yes	Plugins required
Orbit [58]	Goldberg, Ilya et al.	Yes	Yes
CODEX	PerkinElmer	Yes	Yes

### 6.1 Non‐Open‐Source Software

The two most commonly used, non‐open‐source software are InForm and HALO.

InForm is a proprietary digital pathology and immunohistochemistry image analysis software developed by PerkinElmer, which usually comes with Vectra Polaris as an analysis platform and is widely used in clinical research. It excels at classifying cellular phenotypes using machine learning algorithms and automatically detecting and segmenting specific tissue types based on tissue morphology. The software also identifies and isolates weakly expressed and overlapping signals from background autofluorescence to obtain low phenotypic expression and is able to quantify staining levels.

HALO is an image analysis software developed by Indica Labs. The software supports image alignment, segmentation, measurement, quantitative analysis, heat map analysis, cell counting and protein expression analysis, as well as cell sorting and filtering capabilities. It features compatibility with a large number of different file formats and can directly process 21 different file formats including JPG, TIF, OME.TIFFND2, MRXS and QPTIFF.

### 6.2 Open‐Source Software

Four commonly used, open‐source software, ImageJ (FIJI) [59], QuPath [57], Cellprofiler [58] and Orbit [60] are discussed here.

The most flexible, widely used and long‐standing biomedical image analysis is ImageJ (FIJI). Initially developed by the National Institutes of Health (NIH), ImageJ provides a series of analysis tools, including optical density analysis, quantitative analysis, morphological analysis and colour analysis, as well as essential image processing functions. In addition, since ImageJ is Java based, it is possible to write macros or plug‐ins for distribution, and many such specialised plug‐ins exist for public use.

QuPath is an ideal choice for beginners in the field of digital pathology image analysis due to its user‐friendly interface and robust quantitative image analysis capabilities. The software automatically recognises fluorescence and bright field views, enabling the detection of localised cells, positive cells and tissues using its built‐in algorithms. QuPath facilitates image exportation based on preferred pixel settings and is able to integrate with other widely used open tools such as ImageJ, OpenCV, Java Topology Suite and OMERO while supporting the import of various image formats. In addition, QuPath allows the use of several scripting languages such as Groovy, JavaScript and Python to customise the analysis process, enhancing and automating the functionality of QuPath analysis tasks.

CellProfiler is an open‐source MATLAB‐based image analysis software widely used for batch analysis. Its flexible and modular design allows users to set up task flows and pipelines according to their needs and to automate measurements based on tasks. However, a disadvantage is that Cellprofiler is unsuitable for analysing whole slide images (WSI).

The Orbit software quantifies high‐resolution images, particularly large‐sized, WSIs, and identifies regions of interest (ROIs). This software is compatible with ImageJ and CellProfiler, making it an ideal preprocessing tool for batch analysis in CellProfiler. The identified ROI can be further divided into subregions and exported to other compatible software, such as CellProfiler and ImageJ, for additional processing.

### 6.3 Process of Spectral Decomposition and Cell Segmentation

After acquisition, mIHC image processing can be divided into three main parts: spectral unmixing, cell segmentation and feature extraction.

Imaging of fluorescent mIHC slides can be performed using advanced digital WSI scanners such as the Vectra Polaris or the Aperio FL and Hamamatsu NanoZoomer [61]. For example, in the case of Vectra Polaris, a multiphoton confocal microscope is used to capture high‐resolution fluorescence images, with narrow spectral wavelengths captured and imaged, followed by a spectral separation technique to isolate overlapping spectra from different fluorophores. This process allows the instrument to integrate the results of each channel based on the area scanned and imaged [62].

The individual scanning of each channel provides great convenience for subsequent multispectral detection, as the spectral information for each marker is stored in the spectral library of the multiplexed target plate. With each fluorescence channel acquired individually, subsequent analysis steps use software with spectral unmixing algorithms to deconvolve overlapping emission spectra and identify the different wavelengths of light emitted by the fluorophores to separate the different signals.

In image processing, the process of identifying and delineating individual cells is known as cell segmentation. More accurate cell segmentation allows for more accurate subsequent quantification of cell properties. Therefore, cell segmentation is essential for identifying and delineating target cells or regions in mIHC images and several algorithms can be used for this purpose. Segmentation can be based on thresholding, which uses intensity thresholds to separate the target from the background [61], or watershed segmentation, which is based on the intensity gradient of the threshold in the image to finish the segmentation. By interpreting an image as a terrain map and simulating the flow of water from local minima to segment the image into distinct areas representing objects or cells, this method proves effective for objects of irregular shapes and varying sizes [63, 64]. Machine learning and deep learning are widely used in cell segmentation, which can be used to learn the patterns and features of cells by training models on labelled datasets.

After cell segmentation is completed, different types of features can be further extracted to quantify various aspects of cell characteristics, including cell size, shape, intensity, texture and spatial distribution of markers. These features can provide valuable information for downstream analysis and interpretation [65].

### 6.4 Automated Image Analytics

Artificial Intelligence will play an ever‐increasingly important role in assisting pathology analysis. But although almost all image analysis software have advanced image analysis functions such as automatic segmentation, localisation and recognition and even batch automatic analysis, there remain limitations and many hurdles in the application of AI. The tissue staining itself lacks the consistency required for AI to be able to replace the pathologist in being able to recognise cells and cellular structures [66, 67, 68]. In order to achieve a higher level of accuracy, such as using the functionality of these software described in this paper without the aid of programming, it is still crucial for pathologists to assess the results of each batch of images and to adjust the parameters used in the analysis manually [69, 70].

Even if burden on the pathologist is reduced by the use of software enabling batch analysis, visual categorisation of the images from each batch and grouping images with homogenous staining together for batch analysis still requires human input [71]. Typically, in the current stage of the advanced image analysis process, the initial stage of batch analysis performed by the software still requires the pathologist to select an image to use as a reference to adjust parameters such as threshold [72, 73]. However, the steps of immunohistochemistry experiments are tedious, and the results will be affected by a series of factors, such as temperature, humidity, the operating time of each step, light and the pH of the reagent. It is not technically possible to achieve complete consistency during these multiple steps so there is a need for the results to be examined by a pathologist before they can be automated in batches. These software packages are, therefore, usually still limited to the initial groundwork at present.

## 7 mIHC Technology Application

### 7.1 Clinical Application of Tissue Images

mIHC is most commonly used in the clinic to predict a patient's prognosis by analysing the expression of specific cells and proteins in patients samples to predict the development of the patient's disease, thus enabling personalised therapeutic strategies.

The use of immunotherapy in various cancers has successfully transformed the treatment of cancer. With the development of immunotherapy, it is necessary to use efficient methods to detect multiple proteins better to understand the response of the tumour environment to immunotherapy. As a result, mIHC has proven to be highly applicable in cancer immunotherapy and has been successfully applied in many studies of melanoma, lung cancer and various other cancers [74, 75, 76, 77].

A study based on metastatic melanoma showed that the greater the similarity between PD‐1 and PD‐L1 expression in patients, the better the clinical response to anti‐PD1 therapy and, in turn, the overall survival of metastatic melanoma patients treated with anti‐PD1 was found to be improved, giving justification for the use of biomarkers in helping to guide therapy choices for metastatic melanoma [76].

Assessment of the cellular composition and cellular activation of TME using mIHC and DSP techniques has also been shown to be a prognostic factor in the patients' tumour journey. In a study of non‐small cell lung cancer [78], the spatial distribution of infiltrating immune cells was assessed using mIHC and the degree of infiltration and heterogeneous distribution of infiltrating immune cells in the TME was found to correlate with patient response to immunotherapy and patient prognosis [78].

### 7.2 The Development of Artificial Intelligence Applied to mIHC

Predicting tumour progression and patient prognosis using stained WSI is a common application of AI in pathology. Biomarkers contain a large amount of important information about the response, prognosis or prediction of a particular therapy. Therefore, developing AI algorithms that can serve as a guiding reference for personalised treatments based on information from biomarkers would be beneficial for patient care.

The clinical applicability of AI algorithms is already well established. Sorin et al. used IMC with 35 markers on a 1‐mm tissue microarray core and a pretrained neural network model that combines conventional clinical parameters with spatial cellular information (including measurements of cellular TME communities) to predict recurrence after lung cancer surgery [79]. It uses AI tools to extract clinically meaningful information and features from biomarker‐labelled sections and, in turn, uses algorithms to achieve predictive capability.

In addition, there have been new developments in digital pathology that allow virtual staining of tissue sections using AI deep learning or obtaining corresponding fluorescence images directly from images of tissue sections that have not been experimentally processed or have only been brightfield stained.

A study by Zhang et al. shows that a technique known as computational high‐throughput autofluorescence microscopy with pattern illumination (CHAMP) enables highly efficient virtual staining [80]. By using high‐throughput and label‐free imaging of thick and unprocessed tissues with large surface irregularities, the images can be converted into virtually stained histological images in less than 15 s through unsupervised learning. Numerous studies have also used deep learning convolutional neural network algorithms, trained with extensive modelling, to convert images of unstained or haematoxylin–eosin‐stained tissue sections into fluorescent images after biomarker labelling using a model directly without the need for physical staining [81, 82, 83]. The application of deep learning to histological image analysis has been shown to extract valuable information that is difficult for humans to perceive, meaning that scientists can significantly save tumour tissue sample and reduce the time and cost of processing sections to obtain valuable information.

Despite tremendous progress, current clinical implementation of digital pathology‐based AI algorithms requires more extensive prospective evaluation and testing.

## 8 Discussion

In clinical research, mIHC has many advantages and can provide researchers with a more comprehensive view of the complex tumour microenvironment. However, single IHC remains an important technique for practical use due to its simplicity, low cost and high reliability. In addition, single IHC is also indispensable in the protocol planning and design stages, and can provide meaningful information for concentration testing, staining sequence and related steps in the development of mIHC procedures.

In contrast, multiplex immunohistochemistry requires greater technical skill and more delicate experimental handling than single IHC. However, multiplex immunohistochemistry can detect multiple antigen colocalisation on a single tissue section, providing more comprehensive information while reducing the number of tumour sections used, conserving tumour tissue samples and making full use of limited tumour samples.

To more comprehensively investigate the tumour immune microenvironment, mIHC can be used now to stain up to nine colours (eight targets and one DAPI) [84]. More targets mean that experimental efficiency will be improved and allows for more biomarker information to be presented simultaneously in a single section, which has the advantage that the details of the microenvironmental map can be explored deeper, and great savings in tumour tissue samples. However, it is worth noting that more rounds of removal and re‐staining of fluorescent signals will cause the stability of the previous rounds of stained signals to be gradually affected in the tissue section. Therefore, when five or more rounds of staining are required, more time must be spent in the design phase of the research panel, and repeated protocol improvement will be required to find the most appropriate sequence of antibodies that may minimise the loss of signal [53].

## 9 Conclusion

In conclusion, multiplex immunohistochemistry experiments make it easier to identify multiple molecular biomarkers, and at the same time have great potential in scientific research, aiding in the discovery and mechanism of action of tumour‐related biomarkers and can assist researchers in the exploration of the tumour microenvironment and promote cancer screening, detection and prognosis.

## Author Contributions

**Aria Kaiyuan Sun:** conceptualization (equal), writing – original draft (equal), writing – review and editing (equal). **Song Fan:** supervision (equal), validation (equal). **Siu Wai Choi:** investigation (equal), supervision (equal), validation (equal), visualization (equal).

## Conflicts of Interest

The authors declare no conflicts of interest.

## Data Availability

The authors have nothing to report.
